# High‐efficiency CRISPR/Cas‐based editing of *Phalaenopsis* orchid *MADS* genes

**DOI:** 10.1111/pbi.13264

**Published:** 2019-10-07

**Authors:** Chii‐Gong Tong, Fu‐Hui Wu, Yu‐Hsuan Yuan, Yan‐Ru Chen, Choun‐Sea Lin

**Affiliations:** ^1^ Agricultural Biotechnology Research Center Academia Sinica Taipei Taiwan

**Keywords:** agrobacterium‐mediated transformation, gene family, protospacer‐adjacent motif, transformation strategy

*Phalaenopsis* orchids are popular potted ornamental plants around the world due to their beauty, floral diversity and long indoor blooming period. Orchids are also important in plant research, having tiny, dust‐like seeds, crassulacean acid metabolism photosynthesis, complex deletion of the genes encoding NADH dehydrogenase subunits and mycorrhizal symbiosis.

Previously, we investigated the orchid *MADS* gene family, which encodes DNA‐binding proteins that are highly expressed in floral organs and may be important for flower initiation and development (Lin *et al*., 2016). The *Phalaenopsis MADS* gene family includes more than 50 members (Chao *et al*., 2018). As it is challenging to obtain different combinations of mutants in perennial plants such as *Phalaenopsis* using traditional crosses, there is great interest in developing alternative techniques for gene family studies. Clustered Regularly Interspaced Short Palindromic Repeats/CRISPR‐associated endonuclease (CRISPR/Cas) genome editing provides a convenient tool to obtain null and multiple mutants in nonmodel organisms, may prove useful for breeding and plant research (Li *et al*., 2019).

Here, we used two CRISPR/Cas strategies to generate multiple mutants in *Phalaenopsis equestris MADS* genes. First, using a vector containing a hygromycin selection marker (*HPTII*) and *SpCas9* genes, we introduced three *MADS* target sites (*MADS44*,* MADS36* and *MADS8*) together in one vector (pYLMADS8_36_44; Ma *et al*., 2015) or separately in individual vectors (P1300_MADS8, P1300_MADS36, P1300_MADS44; Lin *et al*., 2018) to produce single‐guide RNAs (sgRNAs). We used *Agrobacterium tumefaciens*‐mediated transformation (Hsing *et al*., 2016) to transfect explants with pYLMADS8_36_44 (the 3sg1C strategy) or a mixture of P1300_MADS8, P1300_MADS36 and P1300_MADS44 (the 3X1sg strategy). The transformed Agrobacteria were co‐cultured with *Phalaenopsis* explants (Figure 1a), and the explants were incubated on hygromycin medium for selection (Figure 1b, c). We numbered the antibiotic‐resistant explants (3sg1C#1 to 21 and 3X1sg#1 to 21) and incubated the regenerated transformants (e.g. 3sg1C#17‐1 to 3sg1C#17‐4) individually (Figure 1d). The genomic DNA of 3sg1C and 3X1sg transformants was isolated. Specific primers were used to amplify the target genes, *SpCas9*,* sgRNA* and *HPTII*.

**Figure 1:**
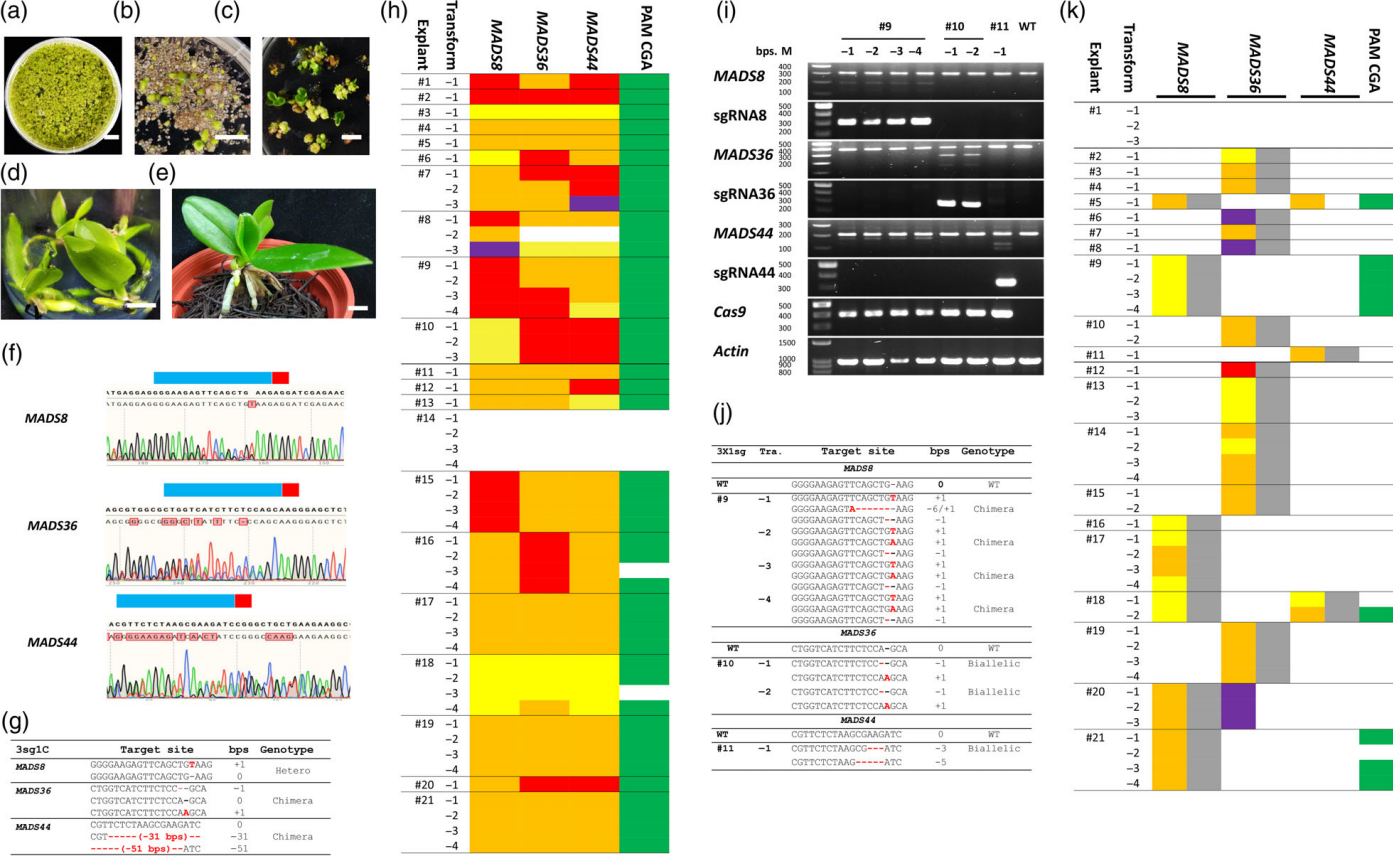
Strategies and results of CRISPR/Cas targeted mutagenesis of *Phalaenopsis MADS* genes. Homozygous, harbours the same edited sequences in both alleles; biallelic, both alleles were edited but the sequences were different; chimera, more than two alleles in the transformants; heterozygous, one wild type and one edited allele. In (g) and (j), letters in red, mutation; – in red, deletion. Numbers in parentheses indicate the number of bases deleted; ‘Hetero’, heterozygous. The coloured blocks in (h) and (k) indicate the genotypes of transformants that were edited in the genes shown above the column. Red, homozygous; orange, biallelic; yellow, chimera; purple, heterozygous and green, mutated in the sequences in *MADS44* that are similar to the *MADS8* target site. (a). One‐month‐old protocorms were incubated with 3sg1C Agrobacteria. Bar = 1 cm. (b). Transfected protocorms were incubated in hygromycin medium. The green protocorms are putative transformed explants. Bar = 1 cm. (c). Transformants with green true leaves. Bar = 1 cm. (d). Rooted 3sg1C#17‐1 to ‐4 incubated in hygromycin medium after 1‐month of subculture. Bar = 1 cm. (e). Two‐month‐old 3sg1C#13‐1 transformant. Bar = 0.5 cm. (f). In the 3sg1C#8‐3 transformant, three *MADS* gene target‐site regions were amplified and sequenced. Blue bar, target site; red bar, protospacer‐adjacent motif (PAM). Multiple peaks start from the sequences near the PAM indicating that there were mutated PCR products. (g). 3sg1C#8‐3 PCR products of three *MADS* gene target‐site regions were cloned, and eight clones from each construct were sequenced to determine the genotype. (h). *MADS8*, *MADS36* and *MADS44* target gene analysis in 3sg1C transformants. The explants are distinguished by lines (#1 to #21). Each row indicates one transformant (‐1 to ‐4). (i). DNA was isolated from each transformant derived from the 3X1sg strategy for PCR, including the target regions of *MADS* genes (*MADS8*, *MADS36* and *MADS44*); sgRNAs of each construct (sgRNA8, sgRNA36 and sgRNA44); *Cas9* and *actin* as an internal control. The *MADS* gene PCR products were combined with wild‐type *MADS* DNA and tested using a T7 Endonuclease I assay. Cleavage of the PCR product indicates the presence of a mutation in this transformant. (j). These mutated *MADS* gene PCR products in (i) were cloned, and eight clones were sequenced for each construct. (k). *MADS8*, *MADS36* and *MADS44* target gene and sgRNA analysis in 3X1sg transformants. The coloured blocks indicate the genotype of transformants that were edited in the genes shown above the column.

The 51 3sg1C transformants were incubated in a growth chamber (Figure 1e). The DNAs of target gene regions were amplified and sequenced (Figure 1f, g, h). All except 3sg1C #14 (no sgRNA) contained the target *MADS* insertion/deletion (indel) mutation(s). Aside from 3sg1C#8‐2, 46 transformants derived from 20 explants contained triple mutants of all three target sites (97.9%, 46/47, Figure 1h). In comparison, a study in *Dendrobium* orchid produced an indel rate of only 10% (15/150) transformates in 15 target sites from five genes and only 33.3% target sites had mutations (Kui *et al*., 2017). Furthermore, 60.0% (12/20, Figure 1h) of explants were homozygous or biallelic for triple *MADS* gene mutations. Thus, all the transformants from these 12 explants were nonchimeric triple *MADS*‐null mutants. This feature makes the technique especially useful for gene editing of long‐juvenile‐phase, heterozygous and vegetative crops such as *Phalaenopsis*.

In the 3X1sg experiment, genotyping of 45 transformants derived from 21 explants indicated that except for 3X1sg#1 (no sgRNA) and 3X1sg#18, which carried two sgRNAs (*MADS8* and *MADS44*), each carried only one sgRNA (Figure 1i, j, k). The numbers of transformants with one sgRNA in each of the indicated genes were 6, 12 and 1 for *MADS8*,* MADS36* and *MADS44*, respectively (Figure 1k). The gene‐editing efficiencies of the sgRNAs were 100% (*MADS8*: 7/7; *MADS36*: 12/12 and *MADS44*: 2/2). The Cas9 codons in pYL‐derived and P1300‐derived transformants have been modified in different fashions—plant‐optimized in pYL‐derived (Ma *et al*., 2015) and human‐optimized in P1300‐derived (Lin *et al*., 2018)—but did not differ in their gene‐editing efficiencies in *Phalaenopsis*. These results indicate that multiple sgRNAs can be combined into a library and transformed to create an edited plant library. The mutants can be selected on the basis of phenotype, and the sgRNA sequenced to identify the gene(s) or DNA region(s) associated with the phenotype.

Compared with pYL‐derived (3sg1C) transformants, P1300‐derived (3X1sg) transformants had a higher proportion of chimerism (33.3%; 14/42, Figure 1k). This contrasts with the situation in protoplasts that were gene‐edited before cell division, which resulted mostly in homozygous or biallelic mutations. Therefore, high‐efficiency gene editing is important for vegetatively propagated crops for which there is no protoplast regeneration system. However, in some CRISPR/Cas9 systems, dicot plants are mutated less efficiently than monocots (Endo *et al*., 2019). Resolving the issue of chimerism in vegetatively propagated dicots will require increased efficiency of both CRISPR/Cas and protoplast regeneration.

Given current public concerns about genetically modified organisms (GMOs), methods to perform gene editing without transgenic gene integration are highly desirable. A recent Agrobacterium‐mediated transient expression experiment in tobacco (*Nicotiana tabacum*) using Cas9 and sgRNA yielded 8% nontransgenic gene‐editing mutants (Chen *et al*., 2018). The transformants from 3X1sg#20 had mutations in the target site of *MADS36* without integration of *MADS36* sgRNA and 3X1sg#5 had mutations in the target site of *MADS44* without integration of *MADS44* sgRNA into their genomes, indicating that nontransgenic targeted mutagenesis occurred in *Phalaenopsis* at an efficiency of 4.8% (2/42, Figure 1k).

The protospacer‐adjacent motif (PAM) neighbouring the target sequence is essential for CRISPR/Cas gene editing. The PAM sequence of SpCas9 is NRG. In *Arabidopsis thaliana*, the average efficiency was 52.7% when following an NGG PAM sequence but 0%–1.1% when following NGA, NGT or NGC (Ge *et al*., 2019). In rice (*Oryza sativa*), the same target site had a 65.5% efficiency when following a TGG PAM but 0% when following TGA, TGT or TGC (Endo *et al*., 2019). Notably, *MADS44* contains a sequence similar to the *MADS8* target sequence but with a CGA PAM. In our 3sg1C experiment, this PAM CGA target site was edited (Figure 1h). In the 3X1sg experiments, however, only the transformants with *MADS8* sgRNA constructs had PAM CGA mutations in *MADS44* (Figure 1k). Mutation to the PAM CGA target site in *MADS44* created a sequence that could act as a *MADS8* sgRNA.

In this study, single, double and triple *Phalaenopsis* mutants can be obtained by different sgRNA construction and transformation strategies, reducing the labour required for transformation. We obtained *MADS*‐null mutants of *Phalaenopsis*, a crop plant with a heterozygous genome and long juvenile period. This protocol has potential applications for gene family studies in other perennial plants.

## Competing interests

The authors declare that they have no competing interests.

## Author contributions

CGT performed *Phalaenopsis* transformation. FHW, YHY and YRC performed molecular biology experiments. CSL designed the experiments, interpreted the data and wrote the manuscript.

## References

[pbi13264-bib-0001] Chao, Y.‐T. , Chen, W.‐C. , Chen, C.‐Y. , Ho, H.‐Y. , Yeh, C.‐H. , Kuo, Y.‐T. , Su, C.‐L. *et al*. (2018) Chromosome‐level assembly, genetic and physical mapping of *Phalaenopsis aphrodite* genome provides new insights into species adaptation and resources for orchid breeding. Plant Biotechnol. J. 16, 2027–2041. 29704444 10.1111/pbi.12936 PMC6230949

[pbi13264-bib-0002] Chen, L. , Li, W. , Katin‐Grazzini, L. , Ding, J. , Gu, X. , Li, Y. , Gu, T. *et al*. (2018) A method for the production and expedient screening of CRISPR/Cas9‐mediated non‐transgenic mutant plants. Hortic. Res. 5, 13. 29531752 10.1038/s41438-018-0023-4 PMC5834642

[pbi13264-bib-0003] Endo, M. , Mikami, M. , Endo, A. , Kaya, H. , Itoh, T. , Nishimasu, H. , Nureki, O. *et al*. (2019) Genome editing in plants by engineered CRISPR‐Cas9 recognizing NG PAM. Nat. Plants, 5, 14–17. 30531939 10.1038/s41477-018-0321-8

[pbi13264-bib-0004] Ge, Z. , Zheng, L. , Zhao, Y. , Jiang, J. , Zhang, E.J. , Liu, T. , Gu, H. *et al*. (2019) Engineered xCas9 and SpCas9‐NG variants broaden PAM recognition sites to generate mutations in *Arabidopsis* plants. Plant Biotechnol. J. 17, 1865–1867. 31070861 10.1111/pbi.13148 PMC6737014

[pbi13264-bib-0005] Hsing, H.X. , Lin, Y.J. , Tong, C.G. , Li, M.J. , Chen, Y.J. and Ko, S.S. (2016) Efficient and heritable transformation of *Phalaenopsis* orchids. Bot. Stud. 57, 30. 28597440 10.1186/s40529-016-0146-6 PMC5430590

[pbi13264-bib-0006] Kui, L. , Chen, H. , Zhang, W. , He, S. , Xiong, Z. , Zhang, Y. , Yan, L. *et al*. (2017) Building a genetic manipulation tool box for orchid biology: identification of constitutive promoters and application of CRISPR/Cas9 in the orchid, *Dendrobium officinale* . Front. Plant Sci. 7, 2036. 28127299 10.3389/fpls.2016.02036 PMC5226938

[pbi13264-bib-0007] Li, B. , Rui, H. , Li, Y. , Wang, Q. , Alariqi, M. , Qin, L. , Sun, L. *et al*. (2019) Robust CRISPR/Cpf1 (Cas12a)‐mediated genome editing in allotetraploid cotton (*Gossypium hirsutum*). Plant Biotechnol. J. 17, 1862–1864. 31055869 10.1111/pbi.13147 PMC6736783

[pbi13264-bib-0008] Lin, C.‐S. , Hsu, C.‐T. , Liao, D.‐C. , Chang, W.‐J. , Chou, M.‐L. , Huang, Y.‐T. , Chen, J.J.‐W. *et al*. (2016) Transcriptome‐wide analysis of the MADS‐box gene family in the orchid *Erycina pusilla* . Plant Biotechnol. J. 14, 284–298. 25917508 10.1111/pbi.12383 PMC11389087

[pbi13264-bib-0009] Lin, C.‐S. , Hsu, C.‐T. , Yang, L.‐H. , Lee, L.‐Y. , Fu, J.‐Y. , Cheng, Q.‐W. , Wu, F.‐H. *et al*. (2018) Application of protoplast technology to CRISPR/Cas9 mutagenesis: from single‐cell mutation detection to mutant plant regeneration. Plant Biotechnol. J. 16, 1295–1310. 29230929 10.1111/pbi.12870 PMC5999315

[pbi13264-bib-0011] Ma, X. , Zhang, Q. , Zhu, Q. , Liu, W. , Chen, Y. , Qiu, R. , Wang, B. , et al. (2015) A robust CRISPR/Cas9 system for convenient, high-efficiency multiplex genome editing in monocot and dicot plants. Mol. Plant. 8, 1274–1284. 25917172 10.1016/j.molp.2015.04.007

